# Beyond job placement: careers for refugees

**DOI:** 10.1007/s10775-023-09579-x

**Published:** 2023-03-30

**Authors:** Nancy Arthur, Mary McMahon, Peyman Abkhezr, Jon Woodend

**Affiliations:** 1 UniSA Business, University of South Australia, Adelaide, SA Australia; 2 The University of Queensland, Brisbane, QLD Australia; 3 Griffith University, Gold Coast, QLD Australia; 4 University of Victoria, Victoria, BC Canada

**Keywords:** Refugees, Career development, Career guidance

## Abstract

In this article, we highlight the Australian country context to advocate for career support that extends beyond initial job placement in a segmented labor market to strengthen refugees’ skills and knowledge and future career development. We address systemic barriers while advocating for access to skilled career development practitioners, whose important expertise as members of multidisciplinary teams could enhance resettlement assistance. Strengthening the preparation of career development practitioners is essential for providing career support to refugees and for building capacity in the provision of resettlement services. We encourage debate within and between country contexts about ways to enhance refugees’ career development.

## Résumé

**Au-delà du placement: Carrières pour les réfugiés**. Dans cet article, nous mettons en avant le contexte du pays australien pour plaider en faveur d'un soutien de carrière qui va au-delà l’insertion professionnelle initiale dans un marché du travail segmenté, afin de renforcer les compétences et les connaissances des réfugiées et leur futur développement de carrière. Nous abordons les obstacles systémiques tout en plaidant pour l'accès à des praticiennes qualifiées en développement de carrière, dont l'expertise importante en tant que membres d'équipes multidisciplinaires pourrait améliorer l'aide à la réinsertion. Le renforcement de la préparation des praticiennes en développement de carrière est essentiel pour fournir un soutien professionnel aux réfugiées et pour renforcer les capacités de prestation de services de réinsertion. Nous encourageons le débat au sein des contextes nationaux et entre eux sur les moyens d'améliorer le développement de carrière des réfugiées.

## Abstract (de)

**Jenseits der Arbeitsvermittlung: Karrieren für Flüchtlinge**. In diesem Artikel beleuchten wir den australischen Länderkontext, um für eine Unterstützung der Berufslaufbahn zu plädieren, die über die anfängliche Arbeitsvermittlung in einem segmentierten Arbeitsmarkt hinausgeht, um die Fähigkeiten und Kenntnisse von Flüchtlingen und ihre zukünftige berufliche Entwicklung zu stärken. Wir befassen uns mit systembedingten Hindernissen und plädieren gleichzeitig für den Zugang zu qualifizierten Fachkräften für berufliche Beratung, deren wichtiges Fachwissen, als Mitglieder multidisziplinärer Teams, die Hilfen zur Integration verbessern könnte. Die Stärkung der Vorbereitung von Fachkräften für die berufliche Entwicklung ist für die berufliche Unterstützung von Flüchtlingen und den Aufbau von Kapazitäten für die Bereitstellung von Integrationsdiensten von wesentlicher Bedeutung. Wir ermutigen zu einer Debatte innerhalb und zwischen den Ländern über Möglichkeiten zur Verbesserung der beruflichen Förderung von Flüchtlingen.

## Resumen

**Más allá de la colocación: Carreras para refugiados.** En este artículo destacamos el contexto del país australiano para abogar por un apoyo a la carrera profesional que vaya más allá de la colocación inicial en un mercado laboral segmentado para reforzar las habilidades y conocimientos de los refugiados y su futuro desarrollo profesional. Abordamos las barreras sistémicas a la vez que abogamos por el acceso a profesionales cualificados en desarrollo profesional, cuya importante experiencia como miembros de equipos multidisciplinares podría mejorar la asistencia al reasentamiento. Reforzar la preparación de los profesionales del desarrollo profesional es esencial para proporcionar apoyo profesional a los refugiados y para crear capacidad en la prestación de servicios de reasentamiento. Alentamos el debate dentro y entre los contextos nacionales sobre las formas de mejorar el desarrollo profesional de los refugiados.

## Introduction

Worldwide, the number of refugees requiring resettlement is rising, which poses many challenges for refugees and the resettlement countries in relation to housing, education, health, and employment (United Nations High Commissioner for Refugees [UNHCR], 2022). Assisting refugees into the labor market is critical for their social and economic participation (Zacher, 2019), developing a sense of purpose, structure, and identity (Fedrigo et al., 2021), and improving physical and mental health outcomes (Dowling et al., 2020). Research on the post-resettlement employment experiences of refugees (e.g., Newman et al., 2018; Richardson et al., 2020; Zacher, 2019) largely focuses on existing barriers for initial entry into the labor market. Specifically, refugees often find that their credentials are unrecognized in their countries of resettlement and/or they are unable to substantiate their credentials and experience due to issues associated with protracted displacement within both their countries of origin and transition (Delaporte & Piracha, 2018; Wehrle et al., 2018). It is undoubtedly valuable for recently resettled refugees to gain local work experience (Tahiri, 2017), but many refugees remain stuck in entry-level, precarious, and lower-paid jobs (Lee et al., 2020) at the bottom tier of the labor hierarchy, commonly known as survival jobs. Often these types of jobs provide limited opportunity for advancement, afford little time for training and advancement, and are not commensurate to refugee workers’ skills and experience (Delaporte & Piracha, 2018), which can negatively impact refugee’s career development and wellbeing (Dowling et al., 2020), as well as increase reliance on social support (Lumley-Sapanski, 2019; Mackenzie Davey & Jones, 2019).

Along with personal wellbeing implications, barriers to refugees’ career development can result in missed social and economic opportunities to build workforce capabilities/capital (Deloitte, 2019; Shergold et al., 2019). Although it is critical to support the initial resettlement of refugees, the long-term career needs of people with refugee backgrounds already living and working in their countries of resettlement should not be ignored (Burhani et al., 2018). The Organisation for Economic Co-operation and Development (OECD, 2021) regards career development as “a fundamental policy lever to help adults successfully navigate a constantly evolving labour market through advice and information on job and training opportunities” (p. 7). The field of career development is well positioned to provide assistance to such workers and could help to address systemic barriers. Similar to domestic born workers and other migrant populations, we contend that refugees’ potential to fully contribute to the labor market may be enhanced through access to ongoing career development support that meets their shifting needs, experience, and skill development (Delaporte & Piracha, 2018).

In order to contribute to the consideration of career development’s role in addressing systemic barriers faced by refugees, in this paper, we explore critical debates and dominant narratives that underpin the career development of refugees in Australia as a call to action for sustainable and meaningful careers across country contexts. First, we review the international and then Australian-based literature on the career development of refugees, then we use the Australian context to provide nuanced examples of the ideas and suggestions for challenging dominant narratives. Gaps in service provision for refugees exist internationally; however, we discuss the Australian context as a point of departure to encourage discussion of the career development needs of refugees in other country contexts. Specifically, we advocate and provide practice-based suggestions for the incorporation of strengths-based, holistic systems approaches to support the career development of refugees, as these approaches consider their emerging and changing life contexts, and their potential contributions to the labor market. Although the examples are Australian-based, our suggestions may have application internationally and encourage inter-country discussions about the best ways to support refugees with their career development.

## Refugees in the world context

Over 26.4 million refugees are forcibly displaced worldwide (UNHCR, 2021a). Of these, the UNHCR identified 1.4 million people are in urgent need of resettlement. Displacement as a result of political instability has been exemplified most recently in 2022 by the war in the Ukraine where, after less than six months, more than five million people moved to neighboring countries (UNHCR, 2022). An emerging issue is that of climate refugees who as a result of climate change (e.g., water-level rises in Pakistan) and subsequent environmental degradation (e.g., desertification) are forced to move from their homes (UNHCR, 2021b). Despite the ongoing and growing global demand for resettlement, between 2018 and 2021, the UNHCR’s resettlement program only supported approximately 157,000 people. Almost all of those were resettled in wealthier, liberal democracies in Western countries, with the top destination countries including Canada, Australia, the United States, the United Kingdom, and Germany. Of those resettled, more than half were children (UNHCR, 2021a). This prioritization of young people in the humanitarian visa programs of receiving countries, along with the very low intake rates, is indicative of a shift in resettlement policies from purely a humanitarian focus to a strategy that incorporates people’s human capital potential for the future. Presumably, younger people will have more years to participate in the workforce and contribute to the economic growth of receiving countries. Although the focus of this discussion is on adults, refugee transitions have implications for future generations and trajectories for education and employment earnings (Baker et al., 2021; Yoshida et al., 2022). Resettlement is complex and extends over several years. It is critical to offer support for refugees beyond the initial settlement period to address persistent barriers and emerging needs and to ensure that they develop careers beyond their initial employment.

## A country context example: Australia

To facilitate critical examination about the career development needs of refugees internationally, we focus on the Australian context as it is consistently one of the top permanent resettlement countries (UNHCR, 2021a) and therefore provides a useful national context for considering the long-term career prospects for refugees in a Western country. However, Australia resettled less than 24,000 refugees between 2018 and 2021 (Australian Government Department of Home Affairs, 2021). Australia’s 2020 goal was to settle up to 20,000 refugees annually, although political and economic circumstances associated with COVID-19 disrupted this target. Similarly, Australia’s 2021–2022 goal to resettle refugees as an important social and economic priority was influenced by its capacity to receive people during pandemic conditions. The long-term implications of these reduced immigration rates have yet to be realized in the post-pandemic world.

### Refugees and employment

Immigration has an important role to play in countries such as Australia, due to declining birth rates and an aging population. Refugees contribute to shaping Australia’s diverse culture, but their workforce participation could be better facilitated to make greater economic contributions (Shergold et al., 2019), while also providing direct outcomes for refugees’ sense of purpose in life, dignity, and wellbeing (Dowling et al., 2020; Fedrigo et al., 2021). A higher intake of refugees in Australia could potentially contribute an additional $37.7(bln) value to the Australian economy over the next 50 years (Deloitte, 2019). Nonetheless, numerous barriers such as language, limited access to local social capital, discrimination and stigma, and lack of recognition of prior qualifications and experiences, can disrupt the integration of refugees after resettlement in Australia, which impacts their successful transition into employment (Khawaja & Hebbani, 2018; Lee et al., 2020; Shergold et al., 2019; Tahiri, 2017; Wali et al., 2018).

The extent to which refugees can contribute to the economy is contingent on their successful transitioning into skilled employment in Australia (Deloitte, 2019; Shergold et al., 2019). Despite 81% of the refugee population being of working age and eager to work (Migration Council Australia, 2019), estimates show that between 38 and 49% of refugees in Australia participate in the workforce. Trends suggest unemployment is rising for this group (Hebbani & Khawaja, 2019). Moreover, of those refugees who participate in the workforce, only 25% are in permanent positions. Research estimates that nearly half of refugees are in jobs that do not use their highest skills or qualifications, or struggle to find employment (Deloitte, 2019). Accordingly, refugees would benefit from utilizing existing skills or upgrading to learn new skills and gain local credentials, but also from opportunities to develop new forms of social capital for employability in the local context (Delaporte & Piracha, 2018; Kivunja et al., 2014). Despite multiple calls to support the inclusion of refugees in the labor market, including a review into integration, employment, and settlement outcomes for refugees and humanitarian entrants in Australia (e.g., Australian Government, 2019; Shergold et al., 2019), there remains a major gap in the literature regarding long-term employment mobility and the influences that support refugees in their career progression over time (Lee et al., 2020). Overcoming systemic barriers necessitates a major shift beyond the initial settlement period and entry-level job placements to fostering the longer-term career development of refugees and their participation in meaningful work.

Importantly, we maintain that the actions of refugees transitioning into the Australian labor market are often over-emphasized with less consideration of the role that could be played by other key stakeholders, including the Australian government and non-government service providers (Arian et al., 2021). For example, the Australian Government’s *Review into Integration, Employment, and Settlement Outcomes for Refugees and Humanitarian Entrants in Australia*’s (Shergold et al., 2019) acknowledged that refugees’ social and economic participation could be advanced through labor market strategies and that services such as “career guidance, mentoring, vocational training, and assistance in accessing non-vocational services such as counselling, work experience, job placements and post-placement support” could address barriers (Australian Government, 2019, p. 8). This discourse suggests a need to critically consider how and where career development services are provided, and by whom, including the roles and qualifications of service providers.

### Employment support for refugees

Typically, employment support in Australia is offered across government departments, or agencies funded by governments. Often, agencies’ funding is conditional on numbers of clients getting into jobs, which makes job placement a priority service. There is less emphasis on long-term career planning, such as the types of jobs (e.g., part time, full time), or their fit with the refugee (e.g., area of interest, experiences, skills), which can funnel refugees into specific sectors (e.g., physical labor; Curry et al., 2018). Refugees’ connection to these services is intended to happen at the point of, or shortly after, resettlement, with the expectation of refugees becoming self-reliant (Dowling et al., 2020). However, in many cases, employment services see refugees return repeatedly as the jobs end, and the risk is that refugees are again funneled into similar sectors or levels of unskilled jobs. Due to the urgency of establishing financial independence for refugees, resettlement agencies often focus on entry-level positions and addressing short-term needs, and there is less focus on long-term career development (Lee et al., 2020; Lumley-Sapanski, 2019). These survival jobs have implications for refugees’ long-term career outcomes such as participation in unskilled or skilled labor positions, contract or full-time employment, and their associated income levels (Lumley-Sapanski, 2019).

In Australia, the discipline of career development has not traditionally been involved in the resettlement and career progression of refugees, and instead, the social and community work fields have predominately provided a range of supports to address immediate needs upon arrival, such as safety, housing, and income support. Formal services may be offered through personnel working in agencies funded by governments, or through informal community organizations with limited funding for staff, relying on volunteers (Goopy et al., 2020). Practitioners and volunteers supporting resettlement have variable backgrounds for providing either employment or career development support. Their contacts with employers may be from a limited pool and there is evidence that refugee clients may be channeled toward pre-existing employer partnerships (Senthanar et al., 2020), rather than considering the best match of their skills and abilities. Unfortunately, it remains the case that gender and occupational sector segregation persists (Ziersch et al., 2022), as do concerns with underemployment, and work conditions such as long hours, low pay, and unhealthy work environments that afford few opportunities for employment mobility (Dowling et al., 2020; Senthanar et al., 2020). Despite concerns about the conditions some refugees face in their employment, evidence suggests that when employers hire refugees, they report being satisfied and open to continuing to hire refugees (Lee et al., 2020). Although the focus of this discussion is not on the role of employers for hiring persons with refugee backgrounds, we acknowledge the importance of employer perspectives in career information and advice (Tahiri, 2017).

In helping to address systemic barriers, we suggest that the discipline of career development could offer support and services, in addition to those already provided, by bringing a set of skills and knowledge and a focus not traditionally applied in refugee resettlement. Adults who are unskilled or who have low-skill levels or are in low-skilled work are disproportionately impacted by the changing labor market and upskilling or retraining may be their best option for finding work and re-engaging in the labor market (OECD, 2021). For younger refugees, career development support could also highlight the importance of a vision for long-term plans and enhance their awareness about the need to develop careers in Australia to successfully navigate the labor market, rather than a mere reliance on entry-level jobs. Career practitioners are well positioned to provide assistance to such workers. For both the refugees and their countries of resettlement, the addition of a career development perspective could enhance the outcomes of resettlement.

## Expanding the focus from initial job placement to careers for refugees

In the next section, we address structural barriers by offering suggestions to prompt critical debate about the current status of career development service provision for refugees and offer strategic directions for the future. Specifically, we advocate for increased access to skilled career development practitioners and upskilling practitioners to work effectively with refugee populations.

In proposing our suggestions, we have considered the OECD’s (2021) guidelines for adult career service provision that relate to provision and service delivery, coverage and inclusiveness, quality and impact, and governance and funding. We highlight the potential contribution of the discipline of career development in resettlement service provision for refugees to expand their employment outcomes beyond initial employment. We are conscious of our positionality and how our worldviews and experiences, such as those of working as academics, practitioners, and researchers in the affluent Western contexts of Australia and Canada, have influenced our perspectives. Although the implications of our suggestions have transferability to different contexts, it is important to consider the unique influences relevant to specific countries in supporting the career development of refugees. We offer the Australian country context to invite debate from persons across countries and disciplines to enhance future directions for policy and practice.

Underpinning our suggestions that follow, in keeping with the OECD (2021) guidelines for lifelong access to career development services for adults, is the fundamental need for a change of focus in service delivery from initial employment placement to a focus on sustainable careers for refugees that offer stability, opportunities to use prior knowledge and skills, and pathways for learning in a new cultural context. We propose that the implications of this change of focus include the following: (a) recognizing diversity within refugee populations, (b) shifting to a strengths-based model, (c) integrating holistic and multidisciplinary perspectives for accessible services, and (d) upskilling of the career development workforce.

### Recognizing diversity of experiences and career development needs

Despite some commonalities among different groups of people with refugee backgrounds, it is crucial to acknowledge their diversity, multiplicity of identities, and life experiences (Bimrose et al., 2016; Yoshida et al., 2022). For example, some people may have spent many years in refugee camps, in detention for seeking asylum, or in transitory countries after being forced to leave their countries (Dowling et al., 2020), while some might have been born in transition, many of whom are considered as stateless persons (UNHCR, 2020). In such contexts, stories of women, people with diverse gender identities and sexual orientations, children and adolescents growing up in transitory and diverse cultures, people with disabilities, and many other groups are essential to be considered for shaping a more inclusive understanding of refugees’ heterogeneity and the need for personalized support. For example, labor market outcomes tend to differ in terms of disadvantage for female in comparison with male refugees, length of time since the displacement from home countries, age, and younger people are generally more adaptable for engaging in education and training (Ivlevs & Veliziotis, 2018; Khawaja & Hebbani, 2018). Customizing and expanding employment and career support requires attention to the diversity of refugees' experiences (Yoshida et al., 2022).

Consistent with the recommendations of the OECD (2021) report, the design of career services must include both general and specialist support for people with diverse identities, tailored to the individual. This implies service provision that addresses commonalities shared by refugees, but also recognizes the unique identities and experiences within that population. For example, whereas some refugees may not have prior access to education or skills training, some have worked as professionals with several years of work experience in their pre-migration countries, while others among them might have acquired education and qualifications in their pre-migration or transitioning countries before final resettlement (Delaporte & Piracha, 2018; Todd et al., 2019). In such cases, support for access to reskilling and upgrading to meet local qualifications is essential for improving employability and to enable transition into more highly skilled and better paid segments of the labor market (Colic-Peisker & Tilbury, 2006).

### Shifting to a strengths-based model

The literature addressing employment of refugees has been both narrow in scope and problem focused. This stems from dominant discourses that represent “international migration as a threat” (Awad & Natarajan, 2018, p. 48) and as an “irregular” act (Awad & Natarajan, 2018, p. 51). Associating forced migration with a “lack of choice” (Verkuyten, 2005, p. 223) has prompted a sympathetic approach to the plight of refugees, but that reaction has mixed consequences. One outcome of such dominant migratory discourses that portray refugees as a homogeneous group with lack of choice, and as traumatized victims, is the application of dominant Western deficit models (Abkhezr & McMahon, 2017; Hutchinson & Dorsett, 2012). The lens through which these deficit models portray refugees is a vulnerability lens (Pesch et al., 2022). Such alignment of refugee support with the dominant Western deficit models further justifies the placement of refugees in the lowest paid and lowest skilled segments of the labor market which has a negative impact on them because their skills and talents remain underutilized and their potential contributions to the economy are diminished (Colic-Peisker & Tilbury, 2006).

A problem-focused approach that views refugees as victims without experiences, valuable skills, and strengths is detrimental (Pesch et al., 2022). It can further internalize stories of victimization and deficiency that inevitably have been told through the screening processes prior to approval of their cases for the ‘UNHCR Resettlement Program’ and the ‘Humanitarian Visa Programs’ of various countries (Australian Government Department of Home Affairs, 2021; UNHCR, 2021a). During the screening process, to clarify their asylum-seeking claims, people must provide detailed accounts (stories) of the experiences that have led to their displacement, review and repeat stories of hopelessness, helplessness, fear, and trauma (Amnesty International, 2013).

In such protracted contexts, opportunities for telling stories of skills, strengths, and abilities are rare (Abkhezr & McMahon, 2017). Later, after arriving in their final country of resettlement and experiencing migration challenges, including barriers to integration and employment, the dominance of stories about self that are restrictive and deficiency-based in nature may further intensify, propagating a ‘culture of disbelief’ (Harris, 2002, p. 4). Given the right approach and environments through provision of a space in which refugees can reconnect with their diverse range of migratory experiences and re-construct stories of skills, strengths, and abilities, service providers can cultivate the sense of ‘agency in waiting’ (Brun, 2015, p. 19) that was culminated in the pre-arrival transitory contexts, toward agentic career actions (Abkhezer et al., 2018). We contend that career development practitioners are well positioned to utilize targeted, tailored, and systemic strengths-based approaches, which would highlight refugees’ sense of agency and enrich their pre-existing stories of career adaptability (Abkhezer et al., 2021). Ultimately, a career development practitioner-facilitated strengths-based approach could contribute to more meaningful, optimistic, and long-term career development (Newman et al., 2018).

### Integrating career development into holistic and multidisciplinary services

Currently, the bulk of the employment advice that refugees receive in their local contexts is provided through government and government-funded agencies run by health and social work professionals and practitioners, as well as volunteers (Kandasamy, 2017). Thus, it is often the case that refugees are not directly or consistently connected with career development practitioners who could introduce them to concepts such as long-term career planning. For refugees, access to career development practitioners may be a trial-and-error process due to a lack of information and understanding about what these services could offer. The career development discipline can raise awareness around who career development practitioners are and the role they can play in supporting refugees’ long-term careers. Including careers practitioners as allied professionals can enhance comprehensive, wrap-around support (Metro Migrant Resource Center, n.d.) and access to other resources and services. Employment services and, arguably, career development services should not be disconnected from a holistic approach to supporting refugee settlement and longer-term integration (Nardon et al., 2021).

Part of this awareness raising involves establishing the career development discipline as a trusted resource, clearly differentiated from immigration officials, social/community work practitioners, and employment services with specialized knowledge beyond social supports (e.g., friends, other refugees). Settlement agencies are often the first point of contact for refugees to engage with the local labor market (Kandasamy, 2017). As refugees establish life in local communities, they often require additional types of support. Staff at settlement agencies have a key role to play in educating their clients about available resources to meet their current and future needs.

Personnel in areas such as education, social work, housing, mental health, and health care are all part of an important network of service providers for supporting refugee clients (Murray et al., 2021; Spinks, 2009). Their knowledge about refugee populations and the specialist expertise of others in an interdisciplinary network can inform holistic and multidisciplinary services for refugees. Encouraging collaborations between service providers underpins building strong networks and shared resources for addressing complex social issues (Holbrook, 2020; Murray et al., 2021), such as prioritizing a common goal of supporting the employment integration of refugees (Metro Migrant Resource Centre, n.d.). Arguably, career practitioners need to be active participants in such networks, to educate other professionals about the functions and value of their expertise, and encourage referral for career support. Career practitioners who work in other community-based settings, such as schools, can work alongside settlement agencies for case management, consultation, and referral (Oliff, 2010). Establishing a network requires community-based career practitioners to build interdisciplinary connections with other professionals who work with refugee clients.

Borrowing from the extensive work in health care and education on interdisciplinary collaboration (e.g., Michalec, 2022; Suter et al., 2009), career development practitioners can advocate for the importance of their roles and learn to effectively communicate the unique benefits and complementarity of their skills. In turn, career development practitioners also need to recognize when the presenting issues of refugee clients are beyond their scope of practice and facilitate referral to appropriate services. This implies that career development practitioners are equipped with the knowledge and skills that they require to work with refugees. Given the limited role that career practitioners have played in the resettlement of refugees, consideration needs to be given to how their specialized skills and knowledge can be utilized in the resettlement of refugees.

## Upskilling of the career development workforce

The competence of career practitioners contributes to the quality and ethical provision of career services (Janeiro et al., 2014; Niles et al., 2019) but it remains the case that “career development lacks the regulation of other professions and the entry pathway is less defined” (O’Reilly et al., 2020, p. 79). One of the pathways for addressing such concerns involves strengthening the expertise and capacity of practitioners who provide employment and careers advice (Burhani et al., 2018).

Most career development training programs focus broadly on diversity and inclusion (see the Professional Standards for Australian Career Development Practitioners, Career Industry Council of Australia [CICA], 2019) and do not specifically address the career development needs of refugees. This gap in training raises serious concerns about the appropriateness of employment and career services for refugees. Career researchers are beginning to investigate the career development of refugees and migrants, and there are initial signs of some evidence-based propositions and considerations for those who work with refugees (e.g., Abkhezr et al., 2018; Abkhezr et al., 2021; Mackenzie Davey & Jones, 2019). Translating this emerging knowledge into improving the preparation of career development practitioners will help to enhance the quality of services provided. Research and theory provide a firm foundation for practice and move it beyond 'good ideas'.

### Theory-informed career practice

Professional standards for career practitioners emphasize theoretical knowledge and connections between theory and practice (e.g., CICA, 2019). Many career theories, however, give little consideration to context and culture (Arthur & McMahon, 2019), and therefore, their relevance to providing career guidance for refugees is not always apparent. Some theories may help practitioners to orient a relevant understanding about career development and refugee transitions and the applications for practice.

At a theoretical level, the complexity of labor market transitions may be understood through the Systems Theory Framework of career development (STF:  Patton & McMahon, 2021). The STF is useful in assisting practitioners to understand the systemic barriers that refugees face and conceptualize their situation (McMahon et al., 2019). It does not identify ‘what works’ in assisting refugees to secure skilled employment. However, recent research (e.g., Abkhezr et al., 2021; Magnano et al., 2021) applied the STF to investigate the career transitions of refugees and made practice recommendations. For example, Abkhezr et al. (2021) demonstrated how complex systemic influences impacted the career adaptability of five young African refugees during their migration journeys. Magnano et al. (2021) applied the STF to investigate the refugees’ social and work inclusion. Both of these studies suggested that systems-based narrative approaches could be useful in supporting refugees’ career development. Thus, the STF could provide a link between theory, research, and practice and serve as a foundational theory for practice.

The Cross-Cultural Life-Career Development (Chen, 2008) framework integrates overarching concepts from several theories that inform career guidance with recent new immigrants, which could be further adapted for working with refugees. A further three theoretical frameworks have potential applications for understanding and supporting the career development potential of refugees during the integration process in destination countries (Zacher, 2019), specifically social-cognitive career theory (Lent et al., 1994; Sheu & Phrasavath, 2019), life-span life-space theory (Super, 1990), and career construction theory (Hartung & Vess, 2019; Savickas, 2002). Related to the latter theoretical orientation, Campion (2018) focused on the link between refugees’ career adaptability and their labor market integration and proposed a job-search model for refugees that incorporates career adaptability, and personal and structural barriers to improve labor market integration.

Massengale et al. (2020) outlined key concepts from the psychology of working theory (Blustein et al., 2019), counselor tasks, and career interventions for supporting refugee populations to overcome systemic barriers and secure decent work. Yoon et al. (2019) offered an example of designing and evaluating a career development program for refugees, based on Hope-Action Theory (Amundson et al., 2020). Another study (Morici et al., 2022) conducted in a European context demonstrates the value of tailored theory-based career counselling for refugees and how these interventions can contribute to improvements in important career management skill such as career adaptability and work search self-efficacy. These examples advance the understanding of how targeted and tailored career development intervention programs benefit refugees through helping them to gain and improve career-related competencies, which contribute to their long-term career development.

Career development practitioners are encouraged to access recent theoretical writing that addresses the importance of culture and context. It is prudent to remember that the majority of theories in the field of career development were developed in Western contexts. Contemporary theorists have recently addressed the applications of their work across cultural contexts (Arthur & McMahon, 2019). Sociological perspectives on career development (e.g., Bimrose, 2019; Hotchkiss & Borow, 1996; Roberts, 2005) emphasize the societal structures and interactions that create advantages or disadvantages for people, such as societal views of gender, race, social class, and their intersections. These perspectives may be useful in addressing the structural barriers many refugees encounter when rebuilding their human and social capital (Delaporte & Piracha, 2018; Murray et al., 2021).

From the perspective of Human Capital Theory (Becker, 1993; Tan, 2014), investing in career services for refugees would be desirable for improving their employability and longer-term employment outcomes. In turn, it is expected that the investment would bring economic returns, as increasing skills recognition and matching individuals to appropriate labor demands would lead to a more productive workforce (Deloitte, 2019). Unfortunately, there continue to be many barriers for the full human capital potential of refugees to be realized. This may be partially explained by labor market segmentation and the necessity of maintaining a labor pool for the least desirable jobs in society, e.g., working in meat-packing plants, cleaning services, and manual labor jobs. Refugees are often placed into low-status, low-skilled jobs with undesirable working conditions when those jobs are rejected by local citizens who have better access to secure and higher-paying jobs (Schenner & Neergaard, 2019). Such practices deskill many refugees when they are hired in jobs that are not commensurate with their prior skills and experience, limiting their potential to shift out of the bottom layer of the labor market. Researchers have also suggested the racialization of refugees from diverse countries and cultural backgrounds is perpetuated in Australia and other countries through the creation of employment niches and precarious integration (Colic-Peisker & Tilbury, 2006). A recent study found that women refugees in Australia are concentrated in lower-skilled and lower-paying occupations, with fewer opportunities for full-time work (Ziersch et al, 2022). These examples suggest that pervasive structural barriers in a segmented labor market restrict the occupational mobility of refugees and limit their human capital potential. While labor market segmentation theory could offer greater insight into the complex factors influencing labor markets and the social and economic costs (e.g., Deakin, 2013; Rubery & Piasna, 2017), a detailed description of this theory is beyond the scope of our article which focuses on the potential contribution of career development services to refugee resettlement.

### Ensuring quality outcomes

The emphasis on securing employment, as quickly as possible, is felt by refugees to support their families and by practitioners to meet organization funding requirements (Refugee Council of Australia, 2010). These ‘survival’ jobs often do not rely on prior education and qualifications, or provide pathways for advancement from unskilled to skilled labor and higher-paying jobs in other segments of the labor market. In working to address structural barriers, we advocate for revised expectations for employment service outcomes that look at the quality of the positions (e.g., full time, ongoing, well paid, relevant), as well as the career readiness of refugees (e.g., capability to navigate job and labor market changes). Indicators of readiness and progress toward those employment outcomes must be taken into account, rather than over-emphasizing the final outcome as job placement, given the variability in preparation that refugees possess at entry points into employment programs.

When funding is tied to job placement, there are fewer resources available to provide follow-up services that would support future career pathways (Senthanar et al., 2020). These structural conditions can lead to a focus on quantity indicators regarding the number of clients in employment while ignoring quality indicators of the suitability or sustainability of employment. Services then become designed around systemic and organizational constraints, rather than the needs of clientele (Mukhtar et al., 2016; Nardon et al., 2021). In contrast, there is evidence to suggest that specialist services, including time to understand the employment needs and capabilities of refugee clients, developing positive and trusted working relationships with staff (Oliff, 2010), and providing long-term services, which includes workplace support, all lead to better employment outcomes (Beadle, 2014; Kyle et al., 2004; Tahiri, 2017).

## Concluding comments

In the context of an increase in the number of refugees seeking resettlement, in this article we focused on strengthening the career development of refugees through advocating to position career development practitioners in the multidisciplinary teams that support them. Assisting refugees to update their qualifications and skills, advance their employment and income prospects, and realize their potential in meaningful and sustainable employment requires an expansion from services that emphasize initial job placement to services that enable their longer-term career development. Access to high-quality career advice and services is crucial to facilitate refugees’ success for transitioning into employment that extends beyond entry-level jobs.

Following on from the points raised by the OCED (2021), there is a need for all adults to have access to career guidance services, including advice and information on employment and training opportunities. The needs of refugees who move between country contexts must be taken into account, including their needs for quality career guidance and employment support. In highlighting the Australian context, we provided an example of destination country-specific debate about the importance of preparing career development practitioners for understanding and working with refugees from diverse cultural and country contexts. In concluding this article, we invite further internal and country-specific debate about the role of career development practitioners as a valuable resource for inclusion on multidisciplinary teams that support initial and ongoing refugee resettlement. Finally, we encourage career practitioners to advocate for structural and systemic changes that will improve career pathways for refugees and also to familiarize themselves with cultural and contextual understandings that prepare them for working with refugee clients.
